# Effect of SiC Reinforcement and Its Variation on the Mechanical Characteristics of AZ91 Composites

**DOI:** 10.3390/ma13214913

**Published:** 2020-10-31

**Authors:** Anil Kumar, Santosh Kumar, Nilay Krishna Mukhopadhyay, Anshul Yadav, Jerzy Winczek

**Affiliations:** 1 Department of Mechanical Engineering, Kamla Nehru Institute of Technology, Sultanpur 228118, India; anilk@knit.ac.in; 2 Department of Mechanical Engineering, Indian Institute of Technology (BHU), Varanasi 221005, India; santosh.kumar.mec@itbhu.ac.in; 3 Department of Metallurgical Engineering, Indian Institute of Technology (BHU), Varanasi 221005, India; mukho.met@iitbhu.ac.in; 4 Membrane Science and Separation Technology Division, CSIR-Central Salt and Marine Chemicals Research Institute, Bhavnagar 364002, India; anshuly@csmcri.res.in; 5 Faculty of Mechanical Engineering and Computer Science, Częstochowa University of Technology, 42-201 Częstochowa, Poland

**Keywords:** metal matrix composites, SiC, AZ91, mechanical characterization, magnesium alloy

## Abstract

In this study, the processing of SiC particulate-strengthened magnesium alloy metal matrix composites via vacuum supported inert atmosphere stir casting process is presented. The effects of small variations in the SiC particulate (average size 20 µm) reinforcement in magnesium alloy AZ91 were examined. It was found that with the addition of SiC particulate reinforcement, the hardness improved considerably, while the ultimate tensile and yield strength improved slightly. The density and *porosity* of the magnesium alloy-based composites increased with the increase in the wt.% of SiC particulates. The tensile and compressive fracture study of the fabricated composites was also performed. The tensile fractures were shown to be mixed-mode fractures (i.e., ductile and cleavage). The fractured surface also disclosed tiny dimples, micro-crack, and cleavage fractures which increases with increasing reinforcement. For the compression fracture, the surface microstructural studies of AZ91 displayed major shear failure and demonstrated the greater shear bands when compared to AZ91/SiC composites, which instead revealed rough fracture surfaces with mixed-mode brittle and shear features.

## 1. Introduction

Magnesium and its composites can supplant steel, aluminium, and plastic-based components due to being ultra-lightweight and having good thermal conductivity. Initially, there was a restriction on the use of magnesium alloys due to the high cost. However, as the cost of magnesium alloys is gradually decreasing, the interest in magnesium alloys has increased.

Magnesium alloy composites are reinforced by various ceramics particles, metals, and carbon nanotubes. Ceramic particles such as SiC, Al_2_O_3_, TiC, MgO, and graphite are mostly preferred reinforcements [1,2,3,4]. The synthesis of the metal matrix composites (MMCs) are fabricated using different technologies such as stir casting [5], powder metallurgy, gas infiltration [6], squeeze casting, spray deposits [7], injection moulding [8] and in situ techniques [9]. There are some other methods of processing magnesium alloys, such as powder metallurgy [10] and friction stir processing [11]. Stir casting is a cost-effective process for the fabrication of magnesium alloy-based MMCs. The manufacturing of Mg alloys and its composites is an immense challenge for scientists and engineers because of their very high affinity towards environmental oxygen. Different casting techniques have been established for Mg alloys and their composites [12]. Stir casting is an efficient casting procedure for the fabrication of particulate-reinforced MMC because of its flexibility, simplicity and lower manufacturing cost [13]. The primary issues identified in stir casting are agglomeration, floating or settling of reinforced particles, and chemical reactions between the reinforcement and matrix alloys. These issues can be avoided by mixing particles in a semi-solid state. Vacuum and inert atmospheres are also generated in the die holder area to prevent oxidation of cast composites. Cast iron was used to make a die in two parts for easy removal of the cast product. The precise method of stir casting process was discussed in our previous work [14].

A new reinforcement method was developed by Wu et al. [15] in which MMCs infiltrate each other within the three-dimensional reinforcements. They concluded that magnesium matrix composite intertwined by stainless steel reinforcement exhibit the better mechanical behaviour. The microstructures of in situ reinforcements Al-Ti-B-C-Ce, Al-Ti-C, and Al-Ti-B, were examined by Tian et al. [16] using a combination of the scanning electron microscope (SEM), X-ray diffraction (XRD) spectroscopy, and TEM. The tensile strength at room temperature (RT) and at 350 °C was enhanced by 19.0%, and 18.4%, respectively. Titanium particulate-reinforced magnesium alloy composites demonstrated better ductility than the ceramic particles. The issue of wettability of pure titanium in pure magnesium was investigated by Kondoh et al. [17]. They concluded that magnesium composites reinforced with 3 wt.% Ti particles showed considerably improved tensile strength and yield strength with good elongation. In situ composites have been developed by adding ceric ammonium nitrate (CAN) into magnesium melt at temperatures of 665 °C and 875 °C. Mechanical behaviour of developed MMCs were evaluated through compression, hardness, and scratch tests [18]. The observations reveal the formation of MgO, CeO_2_, and CeMg_12_ phases in various sizes and shapes. Furthermore, these particles have been attributed enhanced mechanical properties. AZ91 reinforced with four distinct concentrations (0, 0.3, 0.6, 1) wt.% of WS_2_ microparticles have been manufactured using the stir casting method. The XRD and SEM of the equal channel angular pressing (ECAP) deformed samples were examined. A significant improvement was observed in yield strength (YS), ultimate tensile strength (UTS) and elongation [19]. The optimum values of mechanical properties at different % of WS_2_ and passes were determined. The unified effects of the number of ECAP passes and variation in content of SiC particulate were considered by Huang and Ali [20]. It was found that even distribution of matrix grain size and SiC particle segregation depend on the number of ECAP passes. The experimental results revealed that the elastic modulus increased on increasing the reinforcement from 2% to 5%.

Mechanical testing results are essential for comparing the quality, alloy growth, and decrease in non-ferrous materials. Many researchers have fabricated nanocomposites reinforced with Al_2_O_3_ nanoparticles by the ultrasonic-assisted semi-solid stirring method into a cylinder component. Jiang et al. [21] investigated microstructure, mechanical, and wear behaviour of the rheoformed composite parts. The ultimate tensile strength, yield strength, and compression strength are increased with content of TiC in AZ91. Extruded magnesium alloy and composite may exhibit better mechanical properties in comparison with un-extruded alloy and composites like copper and aluminium [22]. Magnesium alloy shows enhanced solidification behaviour over other cast metal, such as aluminium and copper alloys [23]. Casting is the leading synthesis method for the Mg alloy parts which represents 98% of the structural applications of the magnesium [24].

In this study, an indigenous stir casting setup is used to examine the effect of variation (2%, 5%, 8% and 11%) of SiC particulate. The incorporation of an inert atmosphere is also used to provide defect-free cast product. The selection of the size of reinforcement (size and percentage), along with the processing method used in this study has not been attempted by earlier researchers. This research contributes to a new dimension in processing and testing of magnesium alloy composites, which will have wider applications in automobile and aerospace industries. The variation in the size and amount of reinforcement plays a significant role in controlling the mechanical characteristics of the composite. This processing method has an enormous impact on the mechanical behaviour of the composites [25,26,27,28]. Physical tests, such as density and *porosity* measurements, have also been done. The surface morphology of the fracture surface in tensile and compression testing have been examined using FE-SEM.

## 2. Materials and Methods

### 2.1. Selection of Material

The commercial magnesium alloy AZ91 was used as matrix materials in this investigation. The elemental analysis of the alloy was done through optical emission spectroscopy. The main alloying elements of the base alloy are given in Table 1.

**Figure 1:**
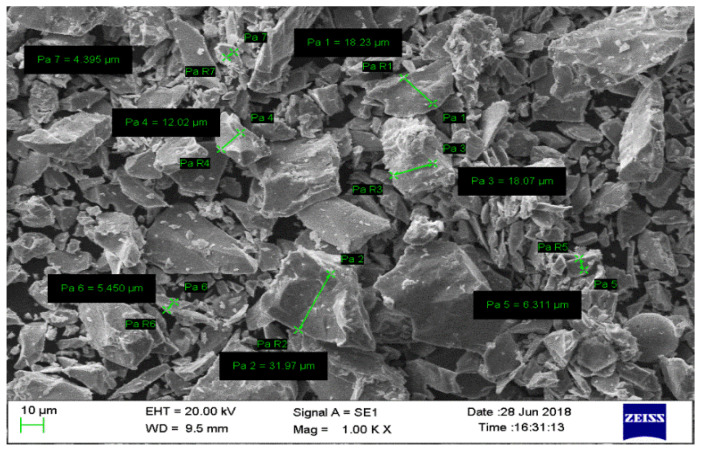
Morphology of SiC particulate.

**Figure 2:**
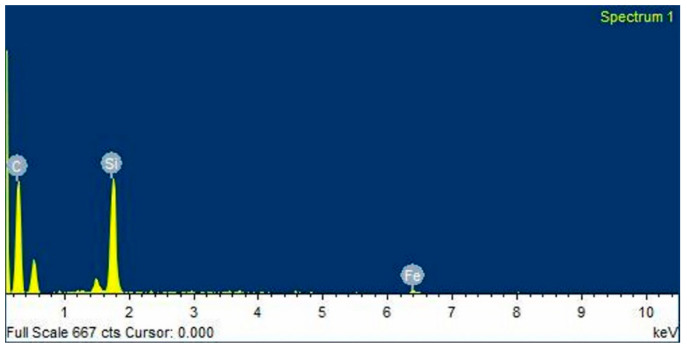
EDAX of SiC particulate.

SiC particulates (average size of 20 µm) were used as reinforcement. Various proportions (2%, 5%, 8% and 11%) of the reinforcement were added to the magnesium alloy AZ91. The basis for the selection of reinforcement is the bonding and wettability between the reinforcement and matrix materials. The morphology of the SiC particulates reinforcement with help scanning electron microscope is given in Figure 1, and Figure 2 shows the EDAX of SiC particulates.

### 2.2. Stir Casting Process

The stir casting method is appropriate for Mg alloy and its composites. The addition and mixing of particles in the matrix were performed using different stirrers. Liquid magnesium has a considerable tendency to be oxidized, and it burns unless proper attention is devoted to ensuring its surface against oxidation. Protection of the liquid Mg with the aid of appropriate flux was used before the application of gaseous shielding. The safest means of shielding liquid magnesium alloy is to make a vacuum followed by impingement of argon gas. In the present study, the Mg alloy-based composite was developed by the stir casting procedure using a vacuum and inert gas. A sound casting product was obtained by the above-described stir casting process. The diameter and length of the cast sample were 3.9 cm and 20.0 cm, respectively.

### 2.3. Density Measurement

Density (ρ) measurements of magnesium alloy (AZ91) and its composites were performed on the polished sample. The density of the AZ91 alloy and its composites was experimentally determined using the Archimedes principle [29]. Filtered water was selected as an immersion fluid. Three samples were arbitrarily selected and were thoroughly weighed both in the air and when fully immersed in distilled water [30]. All weights were measured with an electronic balance (accuracy of 0.001 g).

The following equation was used to calculate experimental density:

(1) $$\rho_{e}=\frac{W_{a}\rho_{w}}{W_{a}-W_{w}}$$

where $\rho_{e}$, $W_{a}$, $\rho_{w}$, $W_{w}$ are the observed density (g/cm^3^), weight of the specimen in air, density of the water and the weight of the specimen in water, respectively.

The theoretical densities of each of the composites were calculated using the rule of mixtures (assuming that there is no Mg/Al/Zn-SiC interfacial reaction) equation, as shown below:

(2) $$\rho_{th}=V_{r}\rho_{r}+(1-V_{r})\rho_{m}$$

where $\rho_{th}$, $V_{r}$, $\rho_{r}$ and $\rho_{m}$ are theoretical density, volume fraction of reinforcement, density of reinforcement and matrix, respectively.

The *porosity* was calculated using the following equation

(3) $$porosity=\frac{\rho_{th}-\rho e}{\rho_{th}}\times 100$$

For theoretical computation of the value of density of the composites, density values of 1.81 g/cm^3^ for the matrix material and 3.216 g/cm^3^ for the SiC particles were used.

### 2.4. X-ray Diffraction (XRD) Studies

The XRD analysis was carried out on the polished specimens [31] of the monolithic magnesium alloy AZ91 and its composites using MiniFlex 300/600 Regaku tabletop XRD diffractometer (Tokyo, Japan) to determine the possible phases available. The samples were exposed to Cu K-α radiation (*λ* = 1.5406 Å) at a scanning speed of 10°/min and step width of 0.020 deg. The scan range was 10–90° with a continuous scan mode. The Bragg’s angle, the value of the interplanar spacing (*d*) and intensity were later matched with the corresponding standard data from Mg, Mg_17_Al_12,_ SiC and other phases.

### 2.5. Mechanical Characterization

#### 2.5.1. Vickers Microhardness

The microhardness (Vickers) test was performed for the entire composite to estimate the homogeneity and uniform distribution of reinforced particles. The hardness values were recorded with different wt.% of SiC reinforcement. The hardness measurements were conducted on LECO’s micro-Vickers hardness machine (St. Joseph, MI, USA) across the polished surface of composites by applying a load of 9.8 N. The pyramidal diamond indenter with a facing angle of 136° was used for indentation tests.

#### 2.5.2. Tensile Test

The tensile testing of particulate-reinforced metal matrix composites was performed to estimate the mechanical properties of vacuum-assisted stir die-cast composites. The tensile samples were prepared from composites with different percentages of SiC particles, as well as commercial magnesium alloy AZ91. The gauge length and diameter of the tensile test sample was 20 mm and 8 mm, respectively, as per the ASTM E8/E8M- 16a standard [32]. The tensile tests were carried out at RT on the Instron-4208 under an initial strain rate of 0.005 s.

#### 2.5.3. Compressive Test

Uniaxial compressive tests at RT were carried out on cylindrical monolithic sample according to ASTM standard E9 [33]. The sample length and diameter were 12 mm and 8 mm, respectively, to make the aspect ratio (l/d) of 1.5. Aimil makes semi-automatic universal testing machine was used to performed compression tests.

#### 2.5.4. Factograph of Tensile and Compressive Tests

Fractured surface studies were conducted on the tensile and compressive fractured specimen’s surfaces of monolithic magnesium alloy and its composites to provide various possible fracture mechanisms operating insight into the sample during tensile and compressive loading. These studies were performed using a ZEISS FE-SEM (Jena, Germany) at different magnifications.

## 3. Results and Discussion

The results of the density measurements of monolithic AZ91 and its composites are presented in Table 2. The measured densities of the composites are remarkably close to the theoretical densities. Thus, near-dense and *porosity*-free composites can be consistently produced using the vacuum-assisted stir casting methodology adopted in the current study. However, as the amount of reinforcement increases, the *porosity* is also found to increase slightly. The density values of the composites are in general higher than those of the monolithic alloys.

Furthermore, the density values are found to increase with increasing content of SiC particulate, due to the presence of the comparatively higher density SiC particulate in the magnesium alloy [34]. The *porosity* also exhibits a gradual increase as the weight percentage of the reinforcement in the composite increases. The increase in *porosity* in composites is due to the increase in micro-voids in the vicinity of massive SiC particles. Similar observations have also been reported by other authors [35,36,37,38,39] with respect to magnesium alloy metal matrix composites reinforced with SiC particulates and fabricated through stir casting techniques.

**Figure 3:**
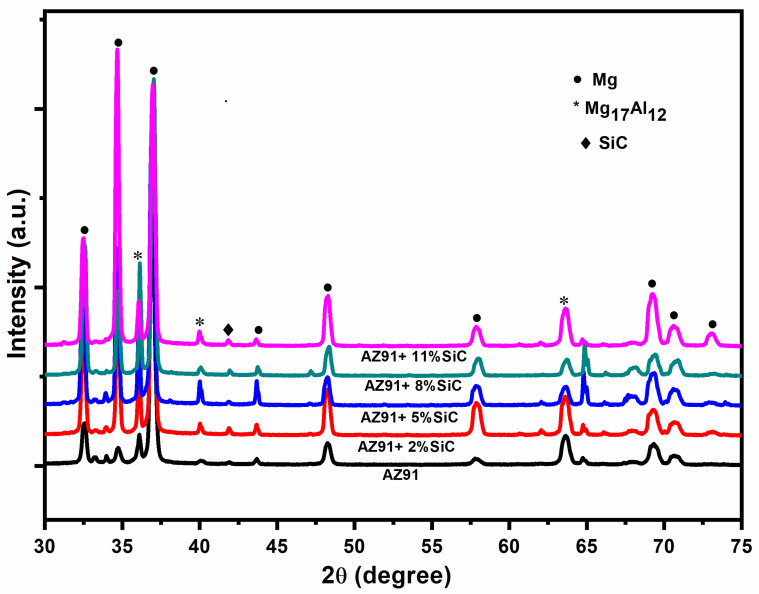
X-ray diffraction pattern of magnesium alloy and its composites containing different amounts (wt.%) of SiC (2%, 3%, 8% and 11%).

Figure 3 shows the X-ray diffraction analysis results corresponding to magnesium alloy AZ91 and its composites with 2, 5, 8, and 11 wt.% reinforcement with SiC particulates. The lattice spacings (d) obtained are matched with that of magnesium, SiCp, and Mg_17_Al_12_ phases based on the data available in JCPDS. This reveals the presence of the magnesium matrix, Mg_17_Al_12_, the intermetallic phases, and the reinforcement SiCp. All the peaks are identified according to the intensity plot of the XRD data. The results confirm the absence of MgO, the formation of which is prevented due to the incorporation of the vacuum and the inert argon gas atmosphere maintained during the melting and casting.

**Figure 4:**
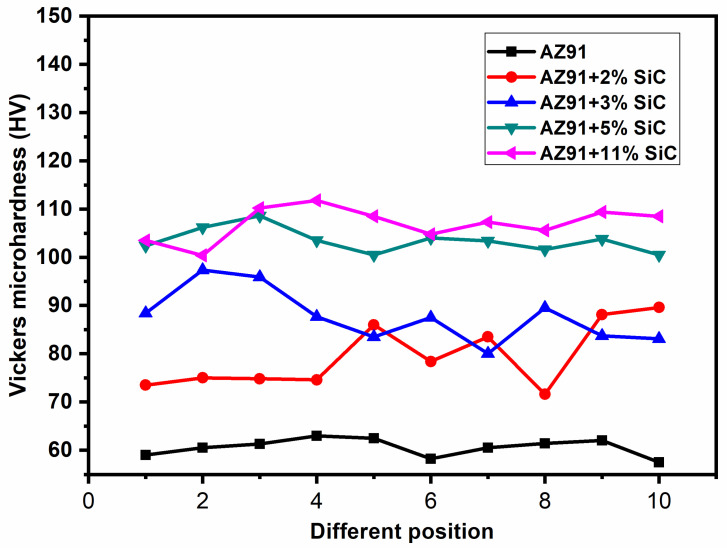
Microhardness data of magnesium alloy and its composites with different (2%, 3%, 8% and 11%) weight percentages at different locations.

A rectangular cross-section sample (15 mm × 30 mm) of the composite samples was used to measure the microhardness. The microhardness data were examined at ten different locations over the cross-section (at the load of 9.81 N) of the AZ91 alloy and SiC particulate-reinforced composites. There is a slight variation in hardness values at different points across the cross-section of the AZ91 alloy and its composites (Figure 4). This establishes the uniform distribution of SiC particles within the magnesium alloy matrix. The variation in hardness in AZ91 alloy is due to the presence of the hard-intermetallic phase in magnesium alloy. This variation is because of the presence of different sizes of reinforcement, as well as slight accumulation of particles in some places. Table 3 shows the average variation in microhardness values of the magnesium alloy (AZ91) and its composites reinforced with SiC particulate with weight percentage variation (2%, 5%, 8%, and 11%). From Table 3, it can be observed that there is an increase in the hardness when the content of SiC reinforcement is enhanced from 2 to 11 wt.%. The Vickers hardness value increases by 28% on the addition of 2% of SiC particulate, and it further increases by up to 80% on addition of 11% of SiC particulate. The observed hardness values of composites with different percentages of SiC (2%, 5%, 8% and 11%) particulate are found to be higher than that of magnesium alloy because of the finer grain size and interactive influence of the presence of SiC particulate, which restricts the localized matrix deformation during the indentation of the composites.

The presence of hard SiC reinforcement will increase the load-bearing capability and also restrict the deformation of the matrix by constraining the dislocation movement [40]. The hardness value of the magnesium alloy composite rises with an increase in the wt.% of SiC particulate reinforcement.

**Figure 5:**
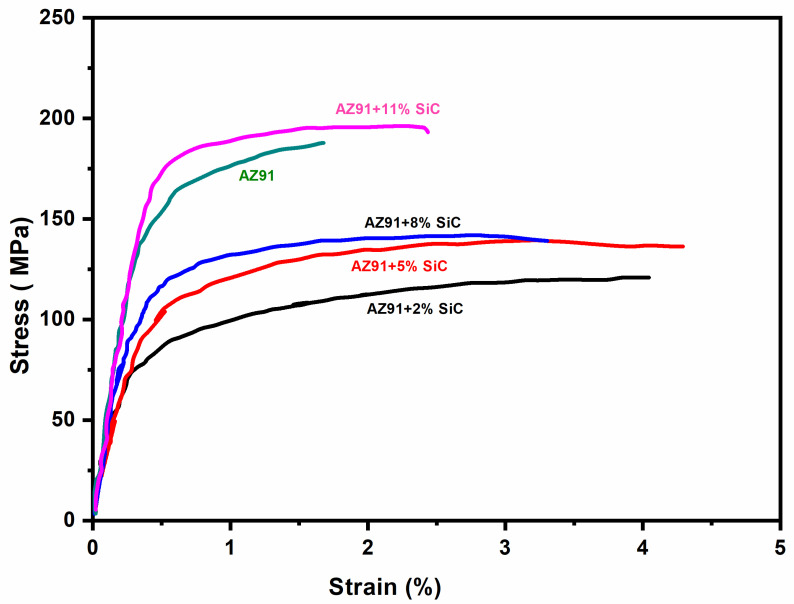
Engineering stress–strain curve of AZ91 and its composites reinforced with SiC particulates.

**Figure 6:**
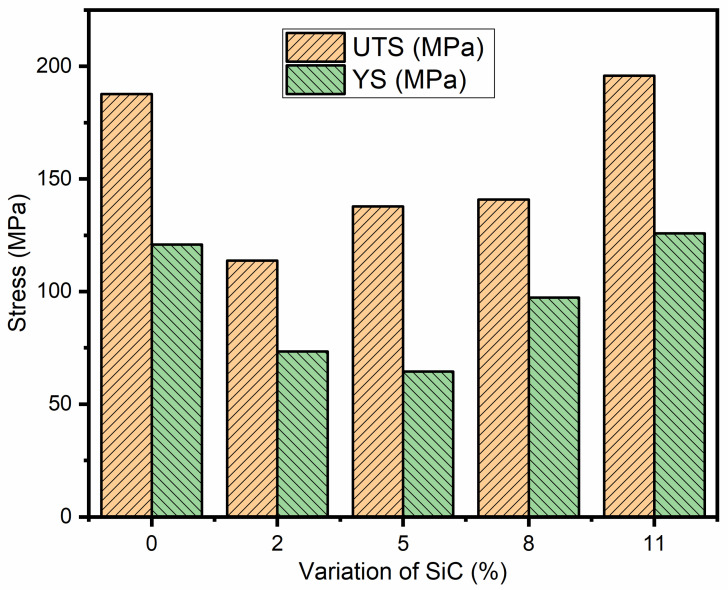
Variation of ultimate tensile strength and yield strength with the variation of SiC particulates.

The ultimate tensile strength (UTS) and yield strength (YS) of the AZ91 and its composites with different weight percentages of SiC (2%, 5%, 8%, and 11%) are given in Table 4. Figure 5 shows the engineering stress–strain curve of magnesium alloy AZ91 and its composites under tensile loading. The graphical bar chart representations of the yield strength and ultimate tensile strength are presented in Figure 6. The ultimate tensile strength (UTS) of AZ91 is found to be 188 MPa. The ultimate tensile strength of the 2% SiC particulate-reinforced composite is lower than that of the magnesium alloy, and it increases when increasing the weight percentage of reinforcements. This result is in agreement with the investigation reported in literature [23]. However, the ultimate tensile strength of 11% SiC particulate-reinforced composites is higher than that of AZ91.

The difference of ultimate tensile strength in AZ91 alloys and SiC particulate-reinforced composite is because of its processing method, which is stir casting in the present study. For the as-cast AZ91 composite, the ultimate tensile strength is generally lower than that of the as-cast AZ91 [23] due to the addition of secondary hard particles and the presence of *porosity*, reducing tensile strength in the as-cast state. Further secondary processing such as extrusion, forging and rolling operation may have enhanced the properties due to the formation of the strong bond between reinforcement, as well as the absence of micro-voids and *porosity*, which are present in the as-cast composites.

The yield strength (YS) of the SiC reinforced composites is observed to improve with an increase in weight percentage of SiC particles in the magnesium alloy (AZ91) composites as depicted in Table 4 and Figure 6. The different strengthening mechanisms may contribute to increased yield strength of the metal matrix composite.

**Figure 7:**
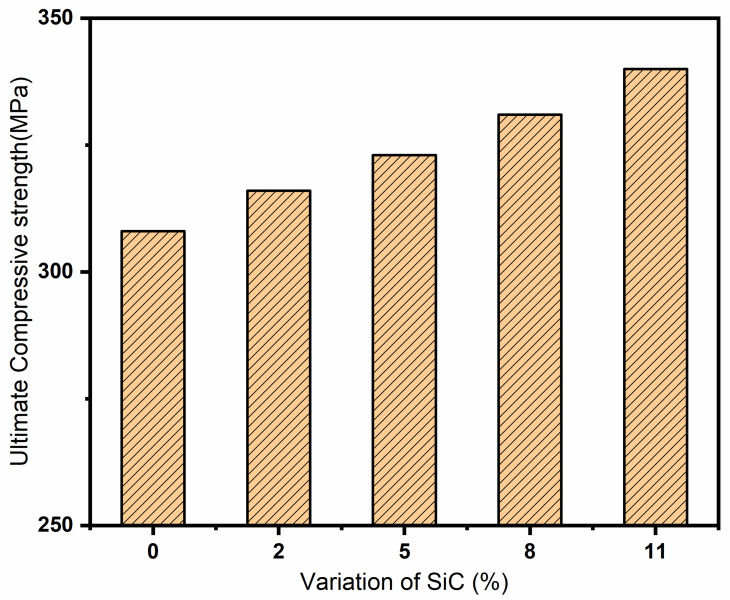
Representation of ultimate compressive strength of alloy and composites with different percentages of SiC.

The compression tests of AZ91 alloy and its composites were performed, and the results are presented in Table 5. The ultimate compressive strength (UCS) of the composite materials reinforced with SiC particulates is higher than that of the unreinforced AZ91 magnesium alloy, as depicted in Figure 7. The UCS of the composites increases further as the weight percentage of SiC particulate increases in the base alloy, AZ91. The UCS of the composites reinforced with different wt.% of SiC increases because of the addition of hard particles in a softer matrix, which increases load-bearing capacity. Significant improvements in ultimate compressive strengths are observed in the SiC particulate-reinforced composites in comparison with the monolithic alloy. The enhancement in ultimate compressive strength may be attributed to the partial closing of the small microscopic cracks and voids during compressive loading. The comparable observation is also reported for SiC_p_ reinforced AZ92 magnesium alloy composites [41]. The compressive strength is increased by 3% on the addition of 2% of SiC particulates, and it is further increased up to 10% on acquisition of 11% of SiC particulate. The compressive strength of AZ91 alloy is sufficiently high, and there is no appreciable increment in compressive strength on the addition of SiC reinforcements.

**Figure 8:**
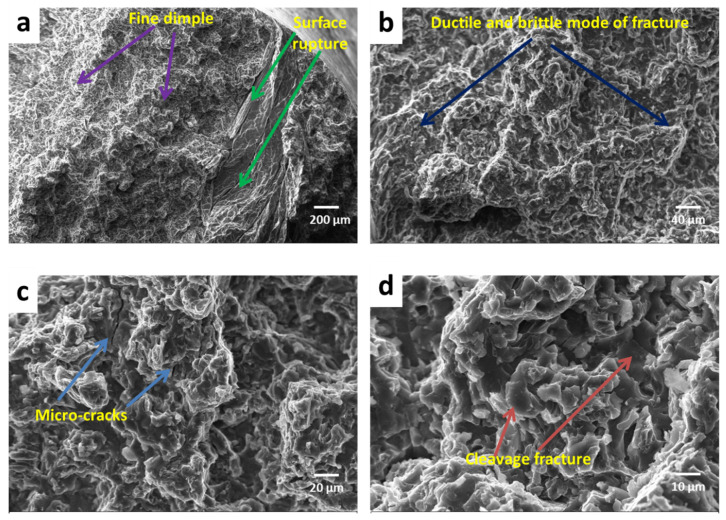
SEM tensile fractograph of cast magnesium alloy AZ91 at (**a**) 100×, (**b**) 500×, (**c**) 1000× and (**d**) 2000×.

**Figure 9:**
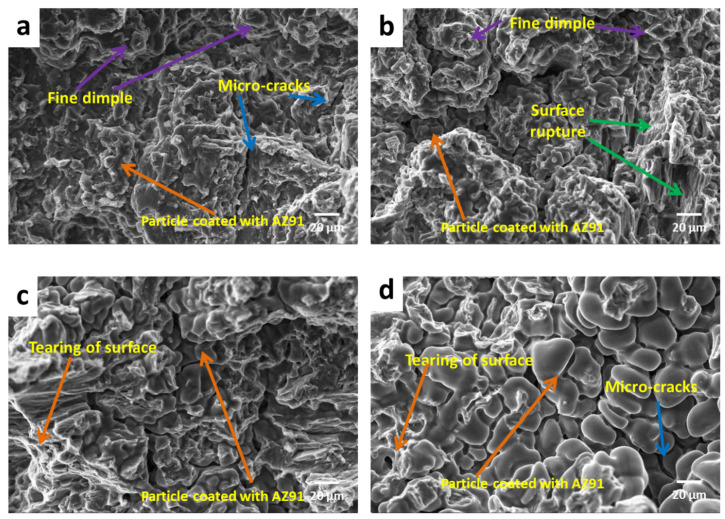
SEM tensile fractograph of cast magnesium alloy AZ91-based composites: (**a**) 2% SiC, (**b**) 5% SiC, (**c**) 8% SiC, (**d**) 11% SiC.

The fracture surfaces of the magnesium alloy AZ91 and as-cast composites reinforced with different wt.% (2%, 5%, 8% and 11%) of SiC particulates were rough, with a normal height variation of 2 mm. Figure 8 shows SEM images of the fracture surfaces of the as-cast magnesium alloy AZ91 at different magnifications. Figure 9 shows SEM images of the fracture surfaces of the as-cast magnesium alloy AZ91-based composites reinforced with different weight percentages of SiC particulates. The main features of the fracture exterior were close to concentrated SiC particulates and agglomeration of SiC particles. The de-bonding of the particle–matrix interface, cracks on the particle, and inter-granular cracks in the matrix were also present. From the fracture surface investigation, it can be seen that fracture occurred via two modes in the case of the composite. In the matrix phase, ductile fracture of the matrix predominantly takes place, in which nucleation of voids plays an important role.

Brittle fracture occurs when the concentration of SiC particles is high. The composite tends to exhibit a more brittle fracture morphology than the matrix alloy. The cracking of the composite is initiated by de-bonding on the interface of the matrix and SiC reinforcement. In the case of the matrix, the fracture takes place by cleavage mode, combined with the ductile feature. However, the composites exhibit mixed-mode fracture (i.e., ductile and cleavage), as well as particle de-bonding. The matrix particle boundary would be mostly controlled by mechanical bonding; hence, when the maximum load is attained, de-bonding of SiC particulates occurs, rather than particle fracturing. The fracture surface characteristics of the composite also shows that the tiny size dimple, micro-crack, and cleavage fracture increases with increase in reinforcement.

In the compression test of AZ91 magnesium alloy and its composites, the fracture occurred nearly 45-degree angle concerning compression test axis and showed dominant shear failure.

**Figure 10:**
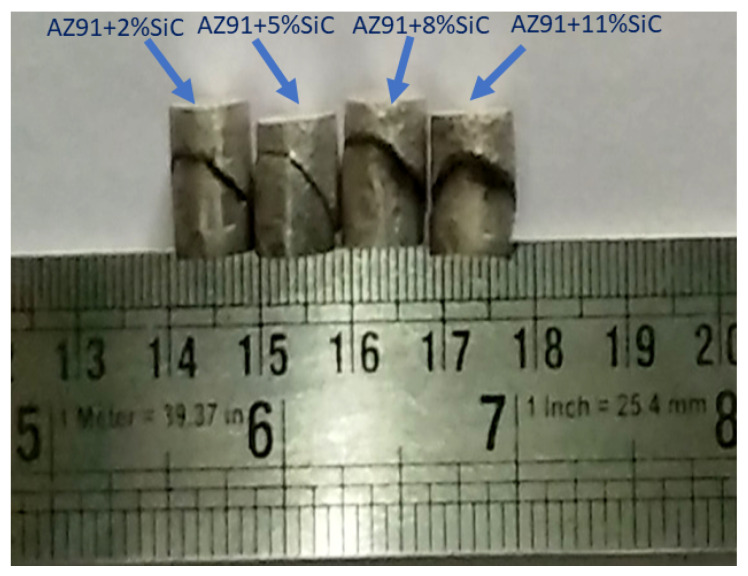
Fractured samples in a compression test.

**Figure 11:**
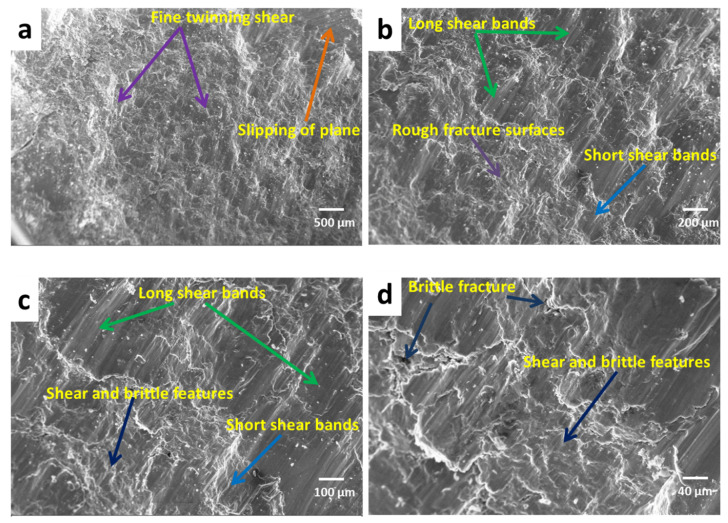
SEM compressive facto-graph of AZ91 at different magnifications: (**a**) 41×, (**b**) 100×, (**c**) 200×, (**d**) 500×.

**Figure 12:**
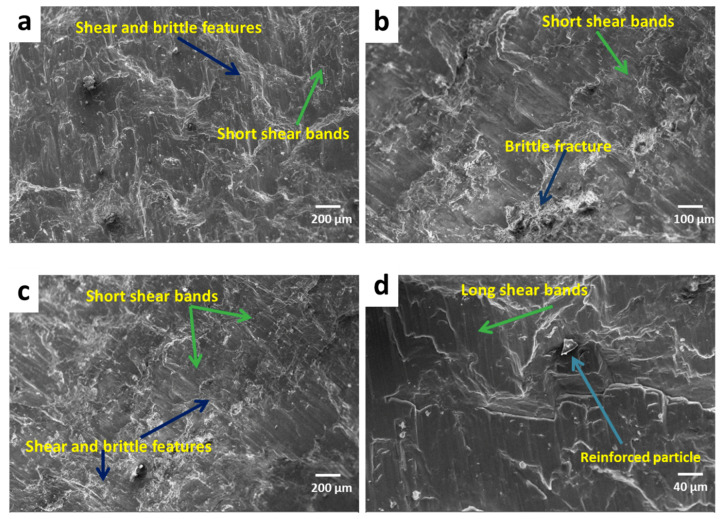
SEM compressive fractograph of AZ91-based composites reinforced with (**a**) 2% SiC, (**b**) 5% SiC, (**c**) 8% SiC, (**d**) 11% SiC.

Figure 10 shows the fractography of the compressive fracture surfaces of AZ91 magnesium alloy-based composites. Figure 11 shows the SEM micrograph of the fracture surface of AZ91 at various magnifications, i.e., 41×, 100×, 200× and 500×. Figure 12 shows the SEM micrograph of the magnesium alloy AZ91-based composites reinforced with a different weight percentage of SiC particulates. The fracture surfaces of AZ91 exhibit major shear failure and show a greater number shear bands when compared to AZ91/SiC composites, which instead show uneven fracture surfaces with mixed-mode, shear and brittle characteristics. Short and long shear bonds are seen in the fractured surfaces, along with the shear twinning mode of plastic deformation. The rough, brittle and shear modes are common fracture modes in magnesium alloys and its composites.

## 4. Conclusions

A technique for preparing defect-free magnesium alloy by casting with improved physical and mechanical properties is presented in this study. The product developed using this casting technique can be used in different engineering applications, such as in aerospace, defence and automobile applications. The following conclusions were drawn based on experiments performed in this study:

- An increase in density was noticed owing to the increase in the amount of high-density reinforcement in the matrix, and an increase in *porosity* was observed because of the increase in micro-voids in the vicinity of the reinforcements.
- The XRD peaks confirmed the presence of Mg, Mg_17_Al_12_ and SiC Phases, indicating that there is no intermetallic bonding between the reinforcement and matrix materials under the recorded processing temperature.
- The Vickers microhardness value was increased by 28% with the addition of 2% SiC particulate, and it was further increased by up to 80% with the addition of 11% SiC particulate. This is because of the increase in load bearing capacity when adding hard particles to soft magnesium alloy.
- The tensile strength was found to initially decrease with the addition of reinforcements (up to 2%), and then increase with the increase in the percentage of SiC particulates (5% to 11%) in magnesium alloys. On increasing the content of the reinforcement, grain refinement occurs, and this led to an increase in ductility.
- The compressive strength increased by 3% on addition of 2% SiC particulate, and it further increased by up to 10% on addition of 11% SiC particulate. The increase in compressive strength can be attributed to the grain refinement, which occurs when increasing the percentage of SiC particulate.
- The composite materials exhibited mixed-mode fracture (i.e., ductile and cleavage). The fracture surface morphology of the composites materials also revealed that the tiny dimples, micro-cracks, and cleavage fractures increased with an increase in reinforcement. This is attributed to the fact that the ductility increases with increasing weight percentage of SiC particulate.
- The compression fractured surface of AZ91 exhibited dominant shear failure and showed more shear bands in comparison to the AZ91/SiC composites, which showed rough fractured surfaces with a mixed mode of shear and brittle features. Strain localization and slightly plastic deformation were observed in composite materials. This is because of the fact that the alloy exhibited more brittleness than the reinforce composites.

Finally, on the basis of the above facts, it can be concluded that the addition of SiC particulates in magnesium alloy enhanced the physical and mechanical properties. Therefore, SiC-reinforced magnesium alloy can be developed for applications in the aerospace, defence and automobile sectors.

## Figures and Tables

**Table 1:** The elemental composition of AZ91.

Mg	Al	Zn	Mn	Si	Other Elements
89.47	8.87	1.02	0.18	0.09	rest

**Table 2:** Density and *porosity* measurements of AZ91 alloy and its composite (AZ91/SiC).

Materials	Theoretical Density (g/cm^3^)	Experimental Density (g/cm^3^)	*Porosity* (%)
AZ91	1.81	1.80 ± 0.02	0.53 ± 0.02
AZ91 + 2% SiC	1.84	1.81 ± 0.01	1.32 ± 0.02
AZ91 + 5% SiC	1.88	1.85 ± 0.02	1.51 ± 0.01
AZ91 + 8% SiC	1.92	1.88 ± 0.01	1.92 ± 0.02
AZ91 + 11% SiC	1.96	1.92 ± 0.01	2.11 ± 0.01

**Table 3:** Vickers microhardness of magnesium alloy and its composite.

Material	Hardness (HV)
AZ91	60 ± 1.8
AZ91 + 2% SiC	77 ± 2.4
AZ91 + 5% SiC	85 ± 3.5
AZ91 + 8% SiC	102 ± 2.5
AZ91 + 11% SiC	108 ± 3

**Table 4:** The ultimate tensile strength and yield strength.

Materials	UTS (MPa)	YS (MPa)	Elongation (%)
AZ91	188 ± 4.5	121 ± 2.8	5 ± 1.1
AZ91 + 2% SiC	114 ± 3.4	73 ± 1.6	10 ± 1.3
AZ91 + 5% SiC	138 ± 2.6	64 ± 1.2	8 ± 0.9
AZ91 + 8% SiC	141 ± 2.7	97 ± 1.4	12 ± 1.2
AZ91 + 11% SiC	196 ± 3.8	126 ± 2.0	7 ± 1.4

**Table 5:** Ultimate compressive strengths of AZ91 alloy and its composites.

Materials	Ultimate Compressive Strength (MPa)
AZ91	308 ± 5.3
AZ91 + 2% SiC	316 ± 2.8
AZ91 + 5% SiC	323 ± 3.5
AZ91 + 8% SiC	331 ± 6.0
AZ91 + 11% SiC	340 ± 2.5
